# Microstructures and Properties of Plasma Sprayed Ni Based Coatings Reinforced by TiN/C_1-*x*_N*_x_*Ti Generated from In-Situ Solid-Gas Reaction

**DOI:** 10.3390/ma10070785

**Published:** 2017-07-11

**Authors:** Wenquan Wang, Wenmo Li, Hongyong Xu

**Affiliations:** Key Laboratory of Automobile Materials of Ministry of Education, School of Materials Science and Engineering Jilin University, No. 5988 Renmin Street, Changchun 130025, China; liwm0905@163.com (W.L.); xuhongy123@163.com (H.X.)

**Keywords:** plasma spray welding, in-situ solid-gas reaction, microstructure and property

## Abstract

The strengthening hard phases TiN/C_1-*x*_N*_x_*Ti were generated by in-situ solid-gas reaction in Ni-based composite coatings prepared using a plasma spray welding process to reinforce the wear resistance of the coatings. The microstructures and properties of the coatings were investigated. The results showed that the coatings mainly consisted of phases such as TiN, C_1-*x*_N*_x_*Ti, TiC, etc. A small amount of CrB, M_7_C_3_, and M_23_C_6_ were also detected in the coatings by micro-analysis method. Compared with the originally pure NiCrBSi coatings, the hardness of the NiCrBSi coatings reinforced by in-situ solid-gas reaction was 900 HV_0.5_, increased by more than 35%. Consequently, the wear resistance of the reinforced coatings was greatly improved due to the finely and uniformly dispersed hard phases mentioned above. The weight losses after wear test for the two kinds of coatings were 15 mg and 8 mg, respectively.

## 1. Introduction

In recent years, metal matrix composite (MMC) coatings have been extensively studied due to the good combination of ceramic hardness and metal toughness. The methods for preparing MMC coatings usually include atmospheric plasma spraying (APS), high velocity oxy-fuel spraying (HVOF), laser cladding, etc. The practical application of MMC coatings have proved that the coatings favor improving the surface properties of materials. However, disadvantages of different preparation methods have limited their further extensive application. For APS and HVOF, the coatings have a mechanical bond with the substrate, which results in poor bonding strength. For laser cladding, the expensive equipment and high maintenance cost will bring heavy burden to customers. Recently, plasma spray welding has attracted more attention from engineers because of its advantages such as metallurgical bond between coating and substrate, better coating density, and desirable coating thickness.

The reinforced hard phases added in the MMC coatings include carbides, nitrides, and oxides such as BN [1], TiN [2,3,4,5,6,7], WC [8], TiC [9], Al_2_O_3_ [10,11], TiO_2_, etc. However, the strengthening effect of added hard phases was often decreased due to their uneven distribution in coatings. The experimental results indicated that the hard phases generated from in-situ reaction process in coatings could overcome this disadvantage. Tekmen [12] and Hoshiyama [13] prepared composite coatings by in-situ plasma spraying which had better properties compared with coatings fabricated by conventional plasma spraying. However, up to now, the literature concerned with composite coatings prepared by in-situ plasma welding is very limited [14]. For the regular hard phases, TiN, TiC, or C_1-*x*_N*_x_*Ti has higher melting point, hardness, and better thermal stability. In this work, the microstructures and properties of Ni-based composite coatings prepared by in-situ plasma welding with Ni60B + Ti powders were investigated. The hard phases such as TiN and C_1-*x*_N*_x_*Ti in the coatings were derived from in-situ gas-solid reaction of nitrogen and metal powders, which introduced a new technique to prepare wear-resistant coatings or layers.

## 2. Experimental Procedures

**Figure 1:**
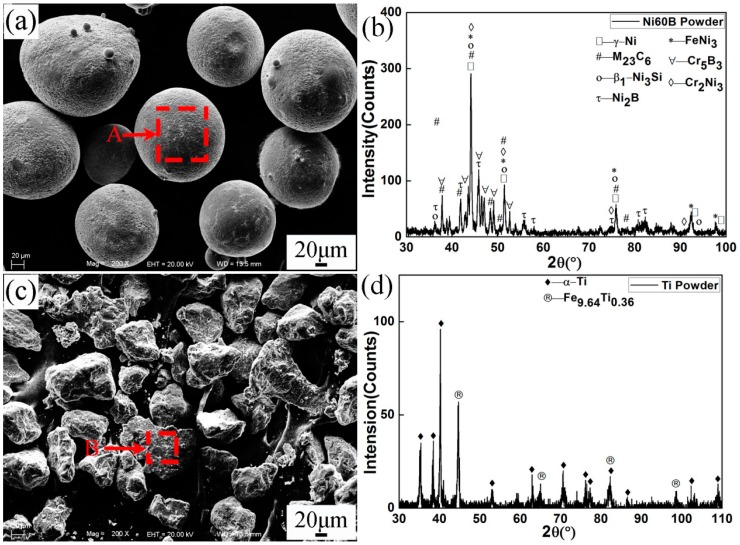
Morphologies and XRD patterns of (**a**,**b**) Ni60B and (**c**,**d**) Ti powders.

Hot work mould steel H13 was chosen as the substrate, and its chemical compositions are shown in Table 1. Ni60B and Ti powders were used as plasma spray welding materials, which were produced by Beijing General Research Institute of Mining & Metallurgy of China. The morphologies, X-ray diffraction (XRD), and energy dispersive spectroscopy (EDS) characterizations of the powders are shown in Figure 1 and Table 2, respectively. It can be seen that Ni60B powder presented in nearly spherical shape, which consisted of γ-Ni, (Cr,Fe)_23_C_6_, β_1_-Ni_3_Si, Ni_2_B, FeNi_3_, Cr_5_B_3_, and Cr_2_Ni_3_. Ti powder presented in irregular shape and mainly consisted of α-Ti and a small amount of Fe_9.64_Ti_0.36_.

In this investigation, 100% Ni60B powder and 85% Ni60B + 15% Ti powders were taken as spray materials, and were labeled as No.1 and No.2, respectively. No.2 powders were mixed by a planetary ball mill (QXQM-4: Company of Mining Machine Equipment, Nanchang, China) for an hour to achieve homogeneous state. The plasma spray welding was carried out using a plasma spray welding system (GAP 2001 DC: Castolin GmbH, Germany) with Ni60B and mixed powders. A plasma spray welding torch operates with two independently adjustable arcs: the transferred arc and the no-transferred arc. The transferred arc was ignited with the aid of a high-frequency voltage and then the no-transferred arc was established between torch and steel substrate, which was used as the energy source for spray welding. Prior to the spray welding, the steel substrate surface was thoroughly cleaned and then rinsed by acetone. The plasma spray welding parameters are given in Table 3. The shielding gases were Ar for No.1 powder and N_2_ for No.2 powder, respectively.

After spray welding, the coating specimens for microstructure study and abrasive test were cut down from the welded H13 substrate. The microstructures and compositions of the coatings were examined by using scanning electron microscope (SEM, EVO18: LaiSe Spectrum Technology Co. Ltd., Shanghai, China) coupled with EDS (Link-ISIS), XRD (Rigaku Corporation, Japan, D/Max 2500PC, λ_CuKα_ = 0.15418 nm, 4°/min). The Vickers micro-hardness of the coatings was tested with a load of 500 g and a duration of 10 s. A wheel grinder (ML-100: JiNan YiHua Tribology Testing Technology Co. Ltd., Jinan, China) was used for wear test of the coatings with abrasive paper 600# under the load of 10 N. The wear resistance of the coatings was evaluated based on their weight loss.

## 3. Results and Discussion

### 3.1. Microstructures of Plasma Spray Welded Coating

**Figure 2:**
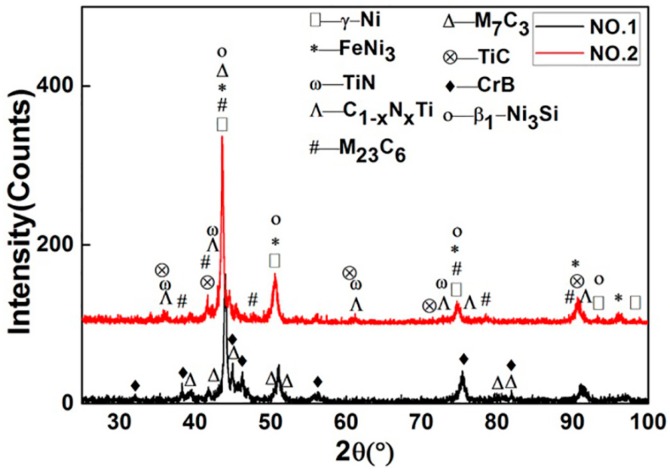
XRD patterns of No.1 and No.2 specimen coatings (0.0 < *x* < 1.0).

Figure 2 shows XRD patterns of specimens No.1 and No.2, respectively. The phases of γ-Ni, FeNi_3_, β_1_-Ni_3_Si, CrB, Cr_7_C_3_, (Cr,Fe)_7_C_3_, Cr_23_C_6_, and (Cr,Fe)_23_C_6_ were identified in the coatings of both No.1 and No.2. From the XRD comparison of No.1 coating and Ni60B powder, new phase CrB was formed but Cr_5_B_3_ and Cr_2_Ni_3_ disappeared after plasma spray welding. In the coating of No.2, a large amount of TiN, C_1-x_N_x_Ti, and a small amount of TiC were found, which derived from gas-solid in-situ reaction. Both TiN and TiC have cubic atomic crystal structure and similar lattice constants. Further, C and N atoms have similar radius. Therefore, element C can be combined with TiN infinitely to form C_1-x_N_x_Ti. The experiment proved that these hard phases greatly increased the wear resistance of the coating. Further study revealed that FeNi_3_ and β_1_-Ni_3_Si decreased in the No.2 coating compared with 85% Ni60B + 15% Ti powder. As a kind of hard and brittle phase, the decrease of β_1_-Ni_3_Si can also improve the wear resistance of the coating.

**Figure 3:**
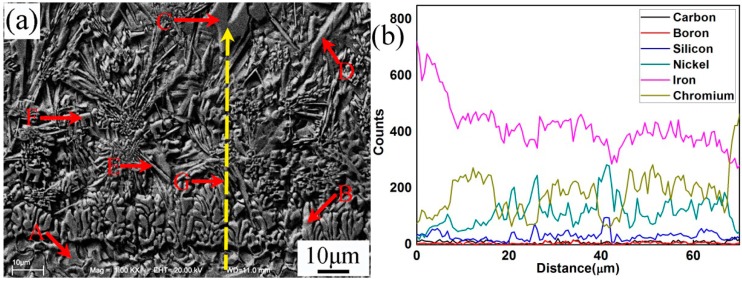
SEM and EDS of interfacial zone of specimen No.1. (**a**) Microstructures of the coating and substrate for specimen No.1 (SEM); (**b**) EDS of interfacial zone of specimen No.1

Figure 3 and Table 4 present the SEM microstructure and EDS analysis of No.1 interface between coating and steel substrate, respectively. It could be observed that the coating and substrate formed metallurgical bonding. The width of the fusion zone (Figure 3a) was about 8–10 μm. According to the EDS line scanning analysis, the substrate was rich in element Fe, which gradually decreased from interface to the top coating. This also indicated that element Fe in substrate diffused into the coating during the process of plasma spray welding. In contrast, the EDS analysis showed that the proportion of elements Ni, Cr, B, and Si in the coating were higher than that in the substrate. From Figure 3a, the coating consisted of columnar grains (B) and irregular carbon precipitates (C, D, E, and F) which were embedded in the matrix. Based on the XRD and EDS results, it could be identified that the matrix of the coating was mainly Ni_3_Si, γ-Ni, and FeNi_3_. The long flakiness shaped carbon precipitates D and irregularly shaped carbon precipitates with holes C were (Cr,Fe)_7_C_3_. However, carbon precipitates D had higher Fe and lower Cr content than that of carbon precipitates C. Both the needle shaped carbon precipitates E and irregular flakiness shaped carbon precipitates F were (Cr,Fe)_23_C_6_, but carbon precipitates E had higher Fe and lower Cr content than that of microstructure F. It could be concluded that the lower Fe content in (Cr,Fe)_7_C_3_ or (Cr,Fe)_23_C_6_ contributed to decreasing the size of the microstructures, which would increase the wear resistance of the plasma spray welded coatings.

**Figure 4:**
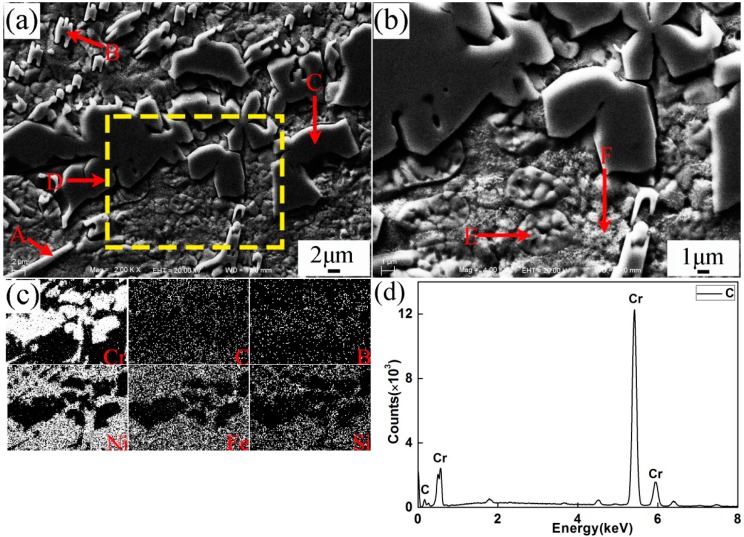
SEM and EDS of coating of specimen No. 1. (**a**) Microstructures of the coating for specimen No.1 (SEM); (**b**) Magnification of D zone; (**c**) The chemical elements in specimen No.1; (**d**) EDS of location C.

Figure 4 presents the SEM microstructure and EDS analysis of the middle part of plasma spray welded specimen No.1. Based on the XRD and EDS results, different shape microstructures were identified as in Table 5. Combined with phase diagram study, it could be concluded that during the process of plasma spray welding, (Cr,Fe)_7_C_3_ and (Cr,Fe)_23_C_6_ first nucleated about 1766 °C and 1576 °C, respectively. The phase γ-(Ni,Fe) precipitated at about 1440 °C and then partly transformed to eutectic microstructures β_3_-Ni_3_Si + γ-Ni at about 1143 °C, which would turn into β_1_-Ni_3_Si + γ-Ni with temperature further lowering. When the welding pool reached about 517 °C, phase γ-(Ni,Fe) at high temperature transformed into phase FeNi_3_, which could only exist at low temperature.

**Figure 5:**
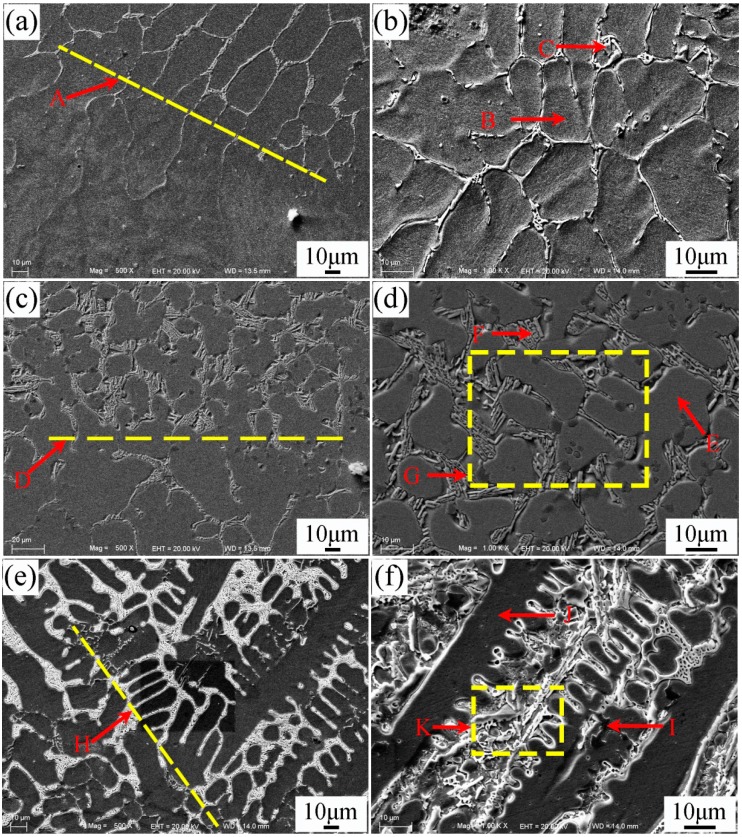
SEM of the interfacial zone of specimen No.2. (**a**) Heat affected zone; (**b**) Morphology of partly melted substrate; (**c**) Interface of melted substrate and coating; (**d**) Cellular grains and boundary precipitates; (**e**) Interface of cellular grains and dendrite grains; (**f**) Dendrite grains of the coating.

**Figure 6:**
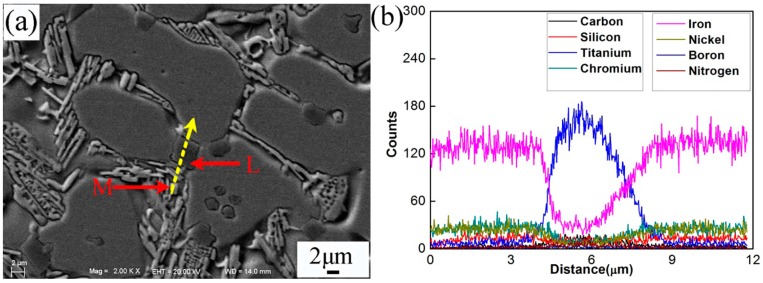
Magnified SEM of point G in Figure 5 and line scan of location M. (**a**) SEM of magnified cellular grains and boundary precipitates; (**b**) EDS of line M.

**Figure 7:**
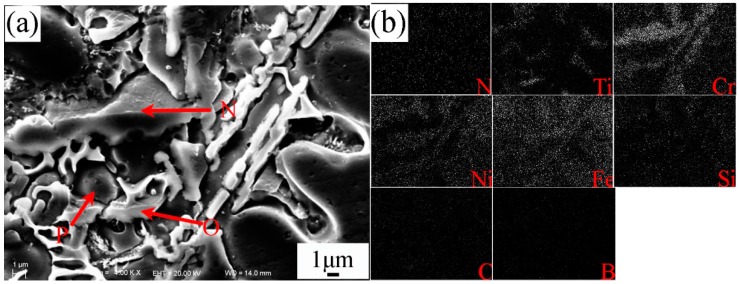
Magnification of area K in Figure 5 and area scan of Figure 5a. (**a**) SEM of magnified dendrite grains of the coating (**b**) The chemical elements in specimen No.2.

Figure 5a–f shows the SEM morphologies of the interfacial zone between substrate and coating of specimen No.2. The magnification and EDS analysis of locations G and K in Figure 5 were presented in Figure 6 and Figure 7, respectively. The EDS analysis results of Figure 5, Figure 6 and Figure 7 are shown in Table 6. The yellow line A in Figure 5a could be seen as the bottom boundary of heat-affected substrate. Figure 5b shows the morphology of partly melted substrate. Due to the thermal effect of the plasma process, the grains B in this region coarsened. The chemical elements Fe, Cr, Ni, and C were detected in the grain boundary precipitates C, which could be attributed to the diffusion of the elements in the coating. The yellow line D in Figure 5c can be seen as the boundary between the melted substrate and coating. Adjacent to the line D, cellular grains could be observed as shown in Figure 5d. The study demonstrated that the content of chemical elements Cr, Mo, V, and C in grain boundary precipitates obviously increased due to the formation of metal carbides. Based on the EDS results, the deep bulk shape microstructure L in Figure 6 was hard phase TiC. This also indicated that the melted spray welding powders mixed with melted substrate in this area. The yellow line H in Figure 5e can be seen as the boundary of cellular grains and dendrite grains, as shown in Figure 5f. According to the analysis results, bulk shape microstructures N and P were (Cr,Fe)_7_C_3_ and FeNi_3_, respectively. The black lump shape titanium carbonitrides I in Figure 5f was C_1-*x*_N*_x_*Ti. Further study had found that there was a large amount of C_1-*x*_N*_x_*Ti and TiC between the secondary dendrite grains. From the EDS results, the content of Fe in partly melted grains, cellular grains, and dendrite grains gradually decreased, but the content of Cr, Ni, and C gradually increased. This was attributed to the dilution effect weakening of the melted substrate on the coating. The microstructures in yellow line K was presented in Figure 7. The analysis proved that carbon precipitates N, O, and intermetallic phase P in Figure 7a were (Cr,Fe)_7_C_3_, TiC + FeNi_3_, and FeNi_3_, respectively. Especially, the formation of TiC in the coating greatly improved the hardness and wear resistance of the coating.

**Figure 8:**
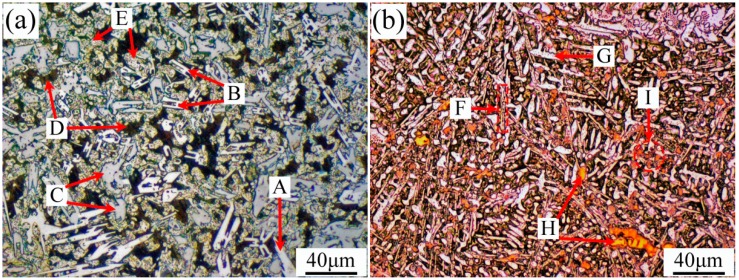
Top coating microstructures of specimens (**a**) No.1 and (**b**) No.2.

The microstructures of the top coatings of specimens No.1 and No.2 are shown in Figure 8. Based on the SEM and XRD analyses, it could be concluded that the long white carbon precipitates A and F were (Cr,Fe)_23_C_6_, “H” shape carbon precipitate B was (Cr,Fe)_7_C_3_, irregular flake-like carbon precipitate C was Cr_23_C_6_, and black matrix D was β_1_-Ni_3_Si + γ-Ni. Further investigation revealed that the clump precipitates of intermetallic phases E and G were FeNi_3_, yellow precipitates of nitrides and titanium carbonitrides H and I were TiN and C_1-*x*_N*_x_*Ti. Compared with the microstructures of specimen No.1, the microstructures of specimen No.2 were fine due to the generation of the small particles of TiN, C_1-x_N_x_Ti, and TiC from in-situ solid-gas reaction, the size of which usually varied from 10–40 μm.

### 3.2. The Properties of Plasma Spray Welded Coating

**Figure 9:**
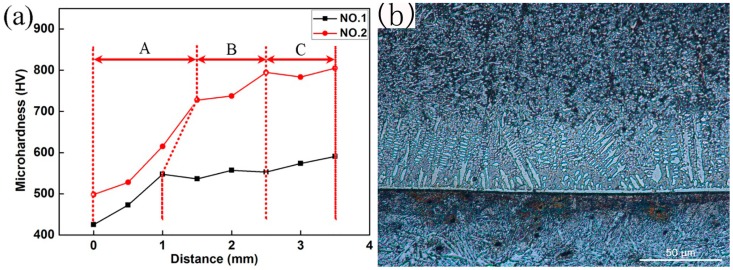
Microhardness of the coating: (**a**) cure of hardness; (**b**) microstructures of the coating and substrate No.1.

The micro-hardness of the plasma spray welded coating is shown in Figure 9. The fusion line was taken as the “zero”, and the hardness of the different points from the fusion line to the top of the coating was measured. Generally, the welded coating can be divided into three parts: the bottom A, the middle B, and the top C. From Figure 9, the bottom A had the lowest hardness, which was mainly attributed to the dilution effect of the substrate material. From the middle part B to the top part C, the hardness of the coating gradually increased because of the rise of the amount of hard phase. The hardness of specimen No.2 was higher than that of specimen No.1, which was attributed to the generation of small particles of TiN, C_1-*x*_N*_x_*Ti, and TiC from in-situ solid-gas reaction.

**Figure 10:**
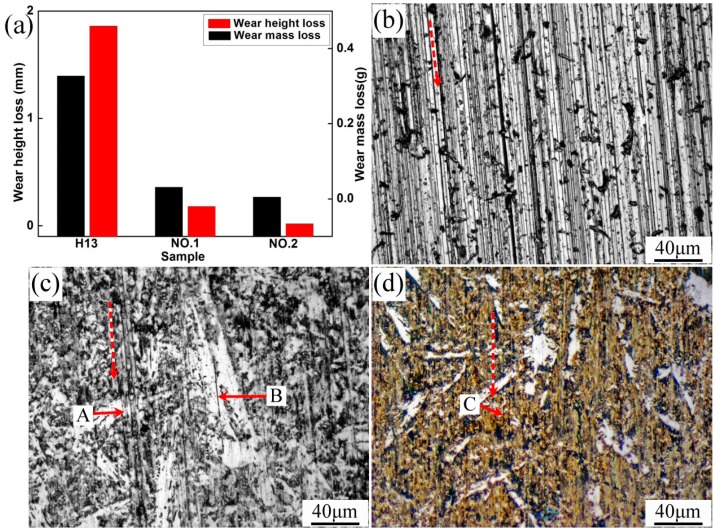
Wear resistance test results: (**a**) weight and height loss; (**b**) H13; (**c**) No.1; (**d**) No.2.

The wear resistance tests for H13, specimens No.1 and No.2 were conducted in this study. The wear resistances of the specimens were evaluated by their height and weight loss before and after tests, as shown in Figure 10. Obviously, specimen No.2 had the best wear resistance. The surface morphologies of the specimens after test are presented in Figure 10b–d. The red dashed lines indicate the direction of the wear test. The plowing mark and plastic deformation were deeper and larger in the surface of specimen H13, but shallower and smaller in the surface of specimens No.1 and No.2 after the wear tests. This also conformed to the wear test results. The investigation demonstrated that fine and hard phases TiN and C_1-*x*_N*_x_*Ti could effectively alleviate the plowing and cutting behavior.

## 4. Conclusions

The metallurgical bonding between the Ni-based coating and steel HB substrate were formed during the plasma spray welding. The microstructures of the coating mainly included (Cr,Fe)_23_C_6_, (Cr,Fe)_7_C_3_, β_1_-Ni_3_Si + γ-Ni, FeNi_3_, TiN, C_1-*x*_N*_x_*Ti, etc. For the coating prepared with powders 85% Ni60B + 15% Ti, a lot of fine hard phases such as TiN, C_1-*x*_N*_x_*Ti, and TiC generated from in-situ solid-gas reaction uniformly dispersed in the coating and greatly enhanced the hardness and wear resistance of the coating. The average microhardness of the coating prepared with 85% Ni60B + 15% Ti powders reached 900 HV_0.5_, and the average wear resistance of the coating was three times that of the coating prepared with 100% Ni60B powder and six times that of the H13 steel substrate.

## Figures and Tables

**Table 1:** H13 Chemical compositions (wt %).

**Cr**	**Mo**	**Si**	**V**	**C**
4.75–5.50	1.10–1.75	0.80–1.20	0.80–1.20	0.32–0.45
**Mn**	**S**	**P**	**Fe**	
0.20–0.50	≤0.03	≤0.03	Surplus	

**Table 2:** Energy dispersive spectroscopy (EDS) analysis of powders.

Element		Ni	Cr	B	Si	Fe	C	Ti
A	at%	46.81	13.72	16.82	7.04	11.83	3.78	-
B	at%	-	-	-	-	9.13	-	90.87

**Table 3:** Parameters of plasma spray welding.

**Non-Transferred Arc (A)**	**Transferred Arc (A)**	**Voltage (V)**	**Powder Feeding Rate**	**Spray Speed (mm/min)**
10	50	33 (45)	30%	35
**Powder Feeding (g/min)**	**Spray Distance (mm)**	**Plasma Gas Ar (L/min)**	**Shielding gas Ar (L/min)**	**Carrier Gas (L/min)**
6.48–6.63	11	0.5	10	4

**Table 4:** EDS results of different points in Figure 3a (at %).

Position	Phase	C	Cr	Ni	Fe	Si	Mo	V
A	γ-(Ni,Fe)	10.53	6.28	9.34	69.63	3.36	0.53	0.33
B	γ-(Ni,Fe)	9.10	11.85	15.96	58.67	3.92	0.16	0.25
C	(Cr,Fe)_7_C_3_	35.49	31.41	-	33.10	-	-	-
D	(Cr,Fe)_7_C_3_	30.53	24.11	7.32	38.04	-	-	-
E	(Cr,Fe)_23_C_6_	18.11	24.24	7.13	50.52	-	-	-
F	(Cr,Fe)_23_C_6_	13.51	32.20	9.22	43.51	0.94	-	-

**Table 5:** EDS results of different points in Figure 4a (at %).

Element	Phase	C	Cr	Ni	Fe	Si
A	(Cr,Fe)_23_C_6_	18.21	45.24	4.33	32.22	-
B	(Cr,Fe)_7_C_3_	34.88	45.93	4.74	14.46	-
C	Cr_23_C_6_	9.32	83.72	1.38	5.58	-
E	FeNi_3_	7.73	-	55.32	24.74	12.21
F	(Ni_3_Si + γ-Ni)	9.09	2.41	59.82	9.80	18.87

**Table 6:** EDS analysis results of different points in Figure 5, Figure 6 and Figure 7 (at %).

Element	Phase	Ti	N	C	Cr	Ni	Fe	Si	Mo	V
B	γ-Fe	-	-	4.11	5.00	3.40	84.51	2.03	0.43	0.51
C		-	-	15.89	7.06	9.54	63.83	2.61	0.45	0.61
E	γ-Fe	0.37	-	11.13	6.70	12.28	65.56	3.43	0.24	0.29
F		7.56	-	10.62	19.19	6.77	49.76	1.07	2.74	2.29
I	C_1-*x*_N*_x_*Ti	48.10	45.83	6.07	-	-	-	-	-	-
J	γ-Fe	1.16	-	13.42	8.85	19.54	52.32	4.38	0.12	0.21
L	TiC	47.72	-	52.28	-	-	-	-	-	-
N	(Cr,Fe)_7_C_3_	-	-	22.38	20.69	15.57	41.36	-	-	-
O	TiC + FeNi_3_	16.24	-	21.42	7.93	15.21	39.19	-	-	-
P	FeNi_3_	1.84	-	10.04	8.43	30.06	45.82	3.81	-	-
